# Numerical Study of Concrete Dynamic Splitting Based on 3D Realistic Aggregate Mesoscopic Model

**DOI:** 10.3390/ma14081948

**Published:** 2021-04-13

**Authors:** Qi Yu, Zhanyang Chen, Jun Yang, Kai Rong

**Affiliations:** 1 State Key Laboratory of Explosion Science and Technology, Beijing Institute of Technology, Beijing 100081, China; yq217@bit.edu.cn (Q.Y.); yangj@bit.edu.cn (J.Y.); kairong@bit.edu.cn (K.R.); 2 China Safety Technology Research Academy of Ordnance Industry, Beijing 100053, China

**Keywords:** SHPB, simulation, dynamic splitting, 3D mesoscopic model

## Abstract

In mesoscopic scale, concrete is regarded as a heterogeneous three-phase material composed of mortar, aggregate and interfacial transition zone (ITZ). The effect of mesoscopic structure on the mechanical behaviors of concrete should be paid more attention. The fractal characteristics of aggregate were calculated, then the geometric models of aggregate were reconstructed by using fractal Brownian motion. Based on the random distribution of aggregates, the concrete mesoscopic structure model was established. And the numerical model was generated by using grid mapping technology. The dynamic compression experiments of concrete under Split Hopkinson Pressure Bar (SHPB) loading verify the reliability and validity of the mesoscopic structural model and the parameters of the constitutive model. Based on these, a numerical study of concrete under dynamic splitting is carried out. By changing the parameters of the constitutive model, the effects of tensile strengths of aggregate, mortar and ITZ on the dynamic tensile strength of concrete are discussed. The results show that the dynamic failure of specimen usually occurs at the interfacial transition zone, then extends to the mortar, and the aggregates rarely fail. However, the increase of strain rate intensifies this process. When the strain rate increases from 72.93 s^−1^ to 186.51 s^−1^, a large number of aggregate elements are deleted due to reaching the failure threshold. The variation of tensile strengths of each phase component have the same effect on the dynamic tensile strength and energy of concrete. The dynamic tensile strength and energy of concrete are most affected by the tensile strength of mortar, following by the ITZ, but the tensile strength of aggregate has almost no effect.

## 1. Introduction

Concrete, as a widely used construction and building material, will be subjected to dynamic loads during its service, such as earthquakes, shocks and explosions [1]. The results of many experiments [2,3,4,5,6] show that the compressive and tensile strength of concrete is significantly sensitive to strain rate. At the same time, as a typical material with different tensile and compressive strength, its tensile strength is far less than the compressive strength. So the tensile strength is considered to be an important material parameter of the tensile and compressive strength different materials. For example, the uniaxial compression failure of concrete is essentially controlled by tensile action perpendicular to the direction of compression load [7]. Therefore, it is necessary to study the mechanical properties of concrete under tensile condition. Separated Hopkinson pressure bar experimental system is widely used to determine the dynamic mechanical properties of materials. It can measure the stress-strain curves of materials at high strain rates (10^2^–10^4^ s^−1^), and is also the most basic experimental techniques to study the dynamic failure process of concrete materials. Nowadays, many scholars have used Split Hopkinson Pressure Bar (SHPB) device to conduct experimental research on dynamic splitting of concrete, and achieved some relevant results [8,9,10,11,12,13].

With the rapid development of computing power, numerical simulation has been widely used as an important scientific method [14,15,16]. Many scholars began to use finite element analysis instead of the SHPB experimental process to study the dynamic response behavior of concrete materials. For example, Jia et al. [17] simulated the SHPB experimental system with a diameter of 50mm and presented a set of calculation methods for concrete Holmquist-Johnson-Cook (HJC) model. Yu et al. [18] analyzed the failure process and stress-strain relationship of concrete with three constitutive models of Karagozian and Case Concrete (KCC), HJC and Continuous surface cap (CSC). Shi et al. [19] used Cowper-Symonds viscoplastic constitutive model considering strain rate effect to simulate the dynamic splitting and crushing process of C75 concrete. All these studies are based on the assumption of macroscopic homogeneity of concrete materials, but the composition of concrete has obvious heterogeneity characteristics. In mesoscopic scale, it is a heterogeneous material composed of randomly distributed aggregate, cement mortar and interfacial transition zone. The non-uniformity of concrete in mesoscopic scale may be the reason for the randomness and discreteness of its mechanical experiment results. The simple assumption of macroscopic uniformity will lead to large deviations between numerical results and experimental results. Wittmann et al. [20] firstly applied the multi-scale research method to study the concrete materials and proposed to use randomly distributed polygons to represent concrete aggregates and established a mesoscopic mechanical model. Zhou et al. [21,22] established a circular random aggregate model and studied the dynamic response behavior of concrete under different stress states. They pointed out that the cohesive interface layer between aggregate and mortar fails at first during compression and has the greatest influence on the failure mechanism and strength of concrete under tensile loading. Wang et al. [23] studied the effects of aggregate shape, aggregate volume fraction and porosity on tensile strength on the basis of elliptic and polygon aggregate models. Sun [24] using X-ray computer tomography (CT) as an effective method of aggregate boundary identification, as well as the equivalent random polygon aggregates of modeling discrete element method (DEM), set up three different porosity of the concrete mesoscopic model and carried out numerical experiments of uniaxial compression, the results show that the crack, under the action of axial force, selectively extends, the minimum principal stress is normally distributed.

In recent years, the establishment method of three-dimensional mesoscopic structure model has gradually developed and matured. Compared with the two-dimensional model, it is closer to the real concrete and can better reflect the deformation and failure process of concrete material. Some scholars began to establish a three-dimensional structural model to study the failure mechanism of concrete at the mesoscopic level. Sadjad et al. [25] used the mosaic and splicing of Voronoi to establish a three-dimensional meso-structure of concrete composed of solid coarse aggregate, mortar, interfacial transition zone and void. The fracture process of concrete under static and dynamic tensile loading is simulated. The results show that the irregular shape of aggregate has an important effect on the crack propagation and final fracture morphology. Zhang et al. [26] used mosaic of Voronoi to generate a random polyhedral aggregate model, and simulated the mechanical behaviors of concrete under quasi-static loading. The damage evolution showed that ITZ had a great influence on the failure of concrete, and the fracture started from ITZ and extended to mortar. Guo et al. [27] regarded concrete as a two-phase material composed of mortar and coarse aggregate, established a three-dimensional random spherical aggregate model, conducted direct dynamic tensile research, and discussed three contact modes between concrete and bars on the basis of experimental and numerical simulation analysis. Sharif et al. [28] established a numerical model of two-phase concrete with homogeneous mortar combined with spherical aggregate, and studied the influence of aggregate grading curve and aggregate volume content on the failure mode. Jin et al. [29] established a concrete mesoscopic model composed of aggregate particles, mortar and ITZ, to study the dynamic compression characteristics of heterogeneous concrete at high temperature and after high temperature, and revealed the influence of high temperature on the macro dynamic compression strength and elastic modulus of concrete. Lv et al. [30] introduced a method to randomly generate the polyhedral aggregate and ITZ. Subsequently, it was applied to the numerical model of concrete specimens by mesh mapping method. They carried out numerical simulation of dynamic compression under the large diameter SHPB loading, and analyzed the waveform characteristics and dynamic failure process of concrete.

Most of the above three-dimensional concrete mesoscopic models simplify the aggregate shape into round, spherical or random convex polyhedron, which deviates from the real aggregate shape. Therefore, we have considered the heterogeneity of the mesoscopic components of concrete in this paper and regard concrete as a three-phase material composed of aggregate, mortar and ITZ. And the three-dimensional mesoscopic structure models of concrete specimens are established according to the fractal characteristics of the real aggregate shape. A SHPB system with a diameter of 120 mm was established to investigate the effect of the tensile strength of three-phase components on the dynamic splitting strengths of concrete at the mesoscopic scale.

The structure of this paper is as follows: After the introduction, the establishment process of three-dimensional mesoscopic geometric model of aggregate and the generation method of ITZ are given in Section 2. Then in Section 3, the mesoscopic numerical model for dynamic splitting of concrete specimens is established, based on the three-dimensional aggregate geometric model, using mesh mapping method. The constitutive model and related material parameters of the three-phase components of concrete are given, and the validity of the numerical model is verified by the published test results. In Section 4, the numerical results of dynamic splitting are discussed. Finally, the main conclusions of this paper are summarized in Section 5.

## 2. Three-Dimensional Mesoscale Model

### 2.1. Generate Aggregate and Mesoscopic Structure

**Figure 1:**
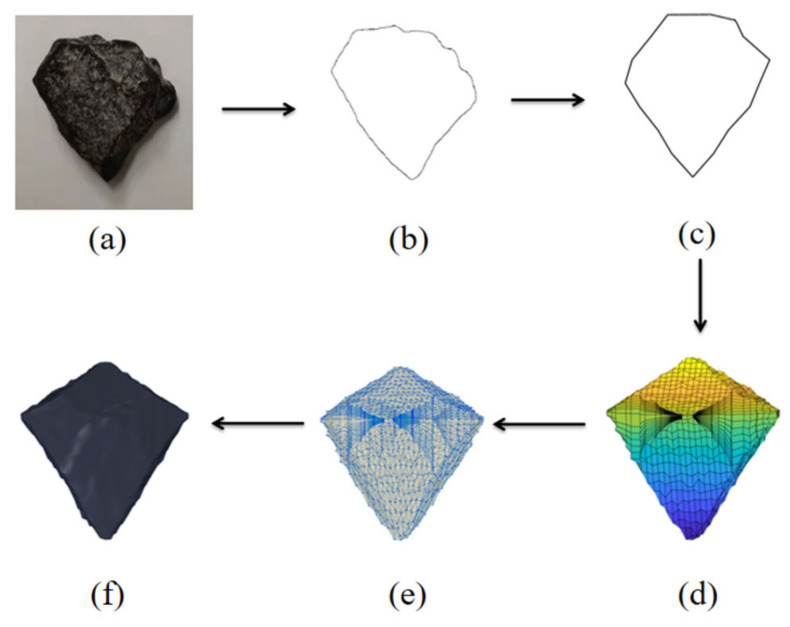
Single concrete aggregate generation, (**a**) the real aggregate; (**b**) contour extraction; (**c**) the reconstructed aggregate; (**d**) closing surfaces; (**e**) Delaunay triangulation; (**f**) mesoscopic model of aggregate.

**Figure 2:**
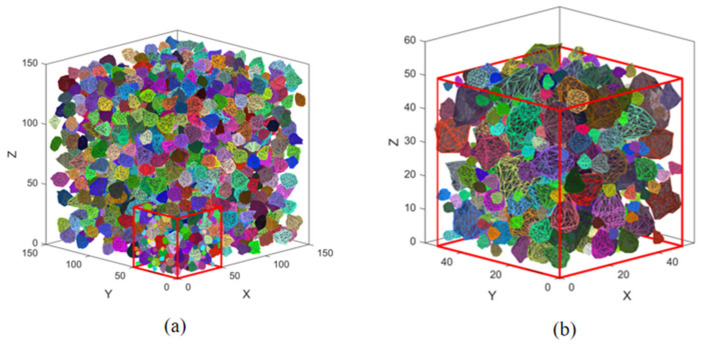
Generated mesoscopic aggregates library, (**a**) aggregates with particle size of 12–20 mm; (**b**) aggregates with particle size of 4–20 mm.

When concrete is simulated at the mesoscopic scale, it is very important to properly describe the geometric shape of the internal composition, that includes the shape characteristics, gradation and spatial distribution of the coarse aggregate. Jin et al. [29] simplified the aggregate into a spherical shape, and Lv et al. [30] made some improvement by approximating the aggregate to a convex polyhedron. However, neither of these two approaches can truly reflect the geometric characteristics of the aggregate in concrete. In this paper, according to the fractal characteristics of realistic aggregate, a mesoscopic model that is more consistent with the real shape is generated. The specific process is as follows:

Firstly, the geometric shape of actual concrete aggregate is calculated by using image recognition technology (gray threshold segmentation) [31]. Fractal characteristics of the aggregates’ geometric shape are counted, and then the aggregate cross-section conforming to the real characteristics of aggregate is randomly generated by fractal Brownian motion model [32]. Then, taking the cross section of an aggregate as the connecting surface, three-dimensional surfaces of the upper and lower parts of an aggregate are constructed respectively and combined into a complete closed surface. The aggregate conforming to the real shape is constructed by controlling the parameters such as the sharp angle, roughness and roundness of aggregate and Delaunay triangulation [33], as showed in Figure 1. The aggregate is generated according to the four gradation standards of 4–8 mm, 8–12 mm, 12–16 mm and 16–20 mm. Finally, the aggregate content in the model was 4%, 8%, 12%, and 16%, respectively, the mesoscale geometric model is shown in Figure 2. Due to the large number of aggregates, Figure 2a only shows the aggregates in the range of 12–20 mm in the entire delivery box, and Figure 2b shows the aggregates in the range of 4–20 mm in the red cube in Figure 2a.

### 2.2. Generation of ITZ

Guinea [34] studied the influence of ITZ layer on the macro-performance of concrete by changing the surface of the aggregate. The research shows that the shape of the aggregate and the strength of ITZ layer have great influence on the macro-fracture performance, and ITZ layer is indispensable in the mesoscopic scale model. If the ITZ layer of aggregate has uniform thickness, the expansion factor can reach the peak at a single point and region, and the whole surface can be transformed, which can meet the demand of generating the interface transition layer.

The expansion factor [35] is defined as follows:

(1) Let $P=(u,v)={\left(x(u,v),y(u,v),z(u,v)\right)}^{T}$ be the $C^{r}$(*r* ≥ 1) like surface defined on the area Ω in the plane *R*^2^ of the parameter (u,v), and B(u,v) be the expansion factor on the same area.
(2) Telescopic center: $O(u_{0},v_{0})=(x(u_{0},v_{0}),y(u_{0},v_{0}),z(u_{0},v_{0}))$.
(3) Stretch coefficient matrix: $D=diag(a_{1},a_{2},a_{3})$, $a_{1},a_{2},a_{3}\in R$, $\Omega_{1}\subset \Omega_{2}\subset \Omega$.
(4) Then the relationship between the curved surface P(u,v) before deformation and the curved surface $P^{\prime }(u,v)$ after deformation is:
  $$P^{\prime }(u,v)=(DB{\left(u,v\right)}^{k}+E)(P(u,v)-O)+O(u,v)\in \Omega$$
  where *E* is the identity matrix; *k* is the fullness index, generally a positive integer, and 5 in the paper.

**Figure 3:**
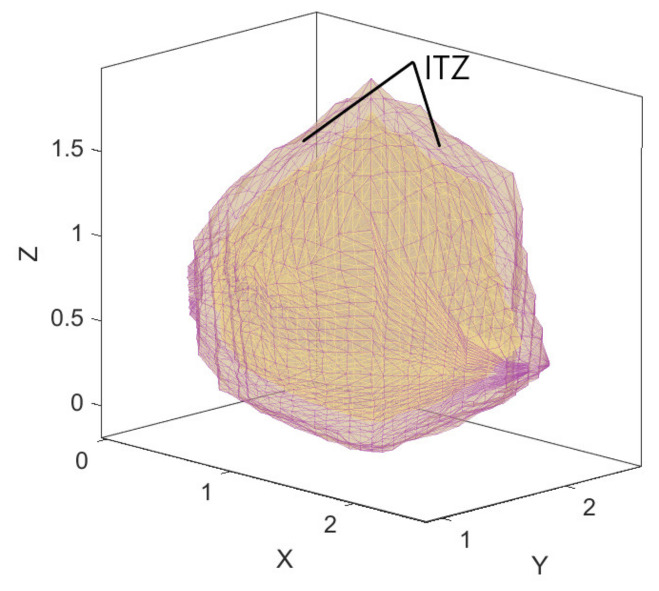
Generation of interfacial transition zone (ITZ).

The expansion factor is used to take the center of the aggregate section as the zoom center, and each vertex coordinate is correspondingly increased by a distance to form a new polyhedral element. The polyhedral element and the corresponding aggregate are ITZ, as showed in the Figure 3.

It is generally accepted that the thickness of ITZ layer is relatively thin, and if the true thickness of ITZ layer is adopted, it will bring great difficulties to calculation efficiency and mesh generation. Depending on reference [36], the thickness of ITZ layer is generally 0.01–0.05 mm. It is difficult to reach the true ITZ thickness in the calculation grid. Therefore, in the process of modeling, the thickness of ITZ layer is set as the size of a calculation grid.

## 3. Establishment and Verification of Numerical Model

### 3.1. Generation of Finite Element Model

After generating the mesoscopic structure model of concrete specimens, it is necessary to transform the model into a finite element calculation model. There are various methods of finite element mesh generation, and the finite element mesh generated by gird mapping method is relatively uniform [37]. In this modeling method, a uniform hexahedron element can be found in aggregate to mortar matrix in the model. The hexahedron element is employed in dynamic calculation, which greatly saves time and improves accuracy in the solution. It should be noted that this approach sacrifices some geometric features of the aggregate, such as sharp corners, smaller convex and concave surfaces. But with the development of computing power, this deficiency will be gradually rectified.

The specific implementation process of the grid mapping method is reproduced below. Firstly, commercial software Hypermesh 14.0 (Altair Engineering Inc., Troy, MI, USA) is used to generate a uniform hexahedron element as a background grid in the space occupied by the geometric model. Then, the mesoscale geometric model is projected onto the background, and, reutilizing the three-dimensional node information, whether the node is in the mortar, aggregate or ITZ layer can be evaluated. After judging the node attributes, it is necessary to identify the material attributes of the unit. The judging criteria and steps are as following:

(1) The initial material properties of all hexahedral elements are transformed into mortar;
(2) When 8 nodes of the hexahedral element have more than *m* nodes located inside the aggregate, the material of the element is judged to be aggregate, and the value of *m* is set to 5;
(3) When there are more than *n* nodes in the remaining mortar unit belonging to the ITZ layer, it is judged that the unit belongs to the ITZ layer, and the value of *n* is also set to 5.

**Figure 4:**
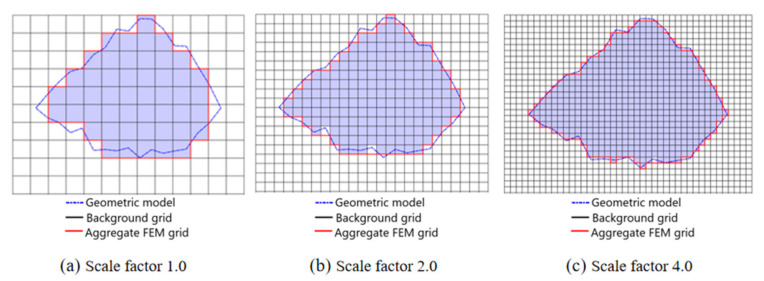
Diagram of grid mapping, (**a**) scale factor 1.0; (**b**) scale factor 2.0; (**c**) scale factor 4.0.

**Figure 5:**
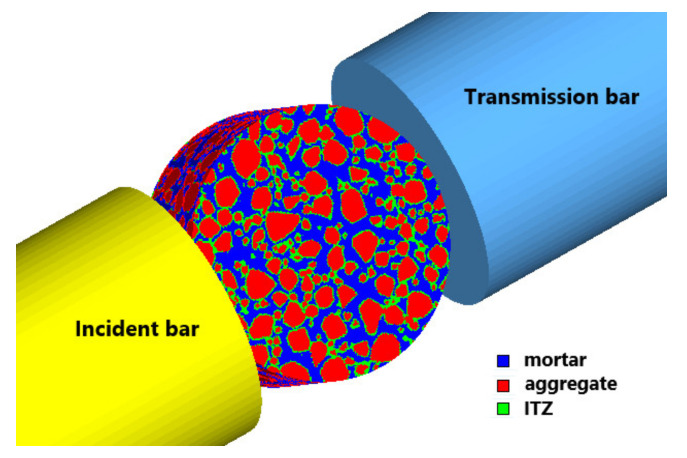
Three-dimensional mesoscale finite element model.

The schematic diagram of the grid mapping method for judging material properties is shown in Figure 4. The black broken line in the figure represents the geometry of the aggregate cross-section generated, and the solid red line represents the cross-sectional shape of the aggregate after mapping. It can be observed that the mesh mapping method can better balance the uniformity of the mesh and the shape of the aggregate. The grid size has a certain influence on the numerical results, and the appropriate grid size can make the calculation results more reliable. As shown in Figure 4, the denser the grid, the closer to the real description of the aggregate contour, but the lower the computational efficiency. Kim et al. [38] set the grid to between 0.1–0.8 mm and found only a small effect on the post-peak performance of concrete. Zhou and Hao et al. [21,22] set the grid as 0.2–0.8 mm and Pedersen [39] as 0.5 mm, both of which can effectively simulate the performance of concrete. According to the previous research results, to reduce the difficulties caused by modeling and calculation, we finally set the grid as 0.8 mm. According to the geometric model generation method of the concrete specimen, the three-dimensional mesoscale finite element model of the concrete specimen is shown in Figure 5. The diameter is 120 mm. The thickness is 60 mm, and the length of the incident bar and the transmission bar is 4 m.

### 3.2. Parameter Selection of Constitutive Model

Adopting the reasonable constitutive model and parameters is very important for the effectiveness of numerical simulation. The incident bar and transmission bar are both aluminum bars, which adopt linear elastic model, with density of 2800 kg/m^3^, elastic modulus of 71 GPa and Poisson’s ratio of 0.33. The KCC (MAT_72 in LS-DYNA) [40] model is used to simulate mortar and ITZ in concrete specimens. Three strength planes are introduced into the material model, namely, the initial yield plane, ultimate strength plane and residual strength plane. Considering the influence of deviating stress invariants on strength, only a few control parameters are input, and the rest of the model parameters can be calculated by LSDYNA_971_R6.1 solver (Livermore Software Technology Corp., Livermore, CA, USA). Because the material properties of ITZ are uncertain, it is generally considered weaker than mortar matrix, and ITZ parameters can be selected according to mortar matrix. The aggregate adopts the HJC (MAT_111 HJC in LS-DYNA) model, and the concrete constitutive parameters of three-phase materials are shown in Table 1, Table 2 and Table 3 [30] respectively.

### 3.3. Validation of Numerical Models

**Figure 6:**
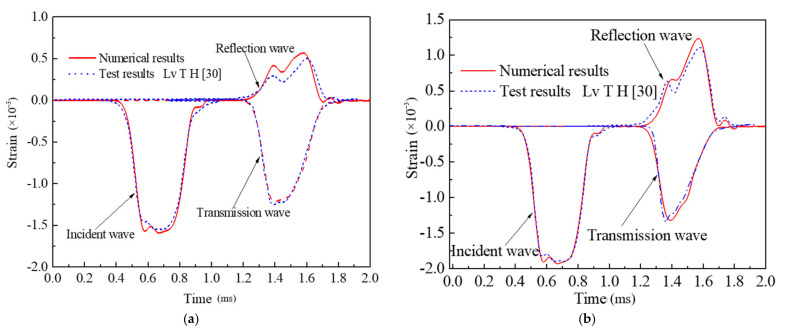
Waveform validation (**a**) impact velocity of 15.33 m/s; (**b**) impact velocity of 18.58 m/s.

**Figure 7:**
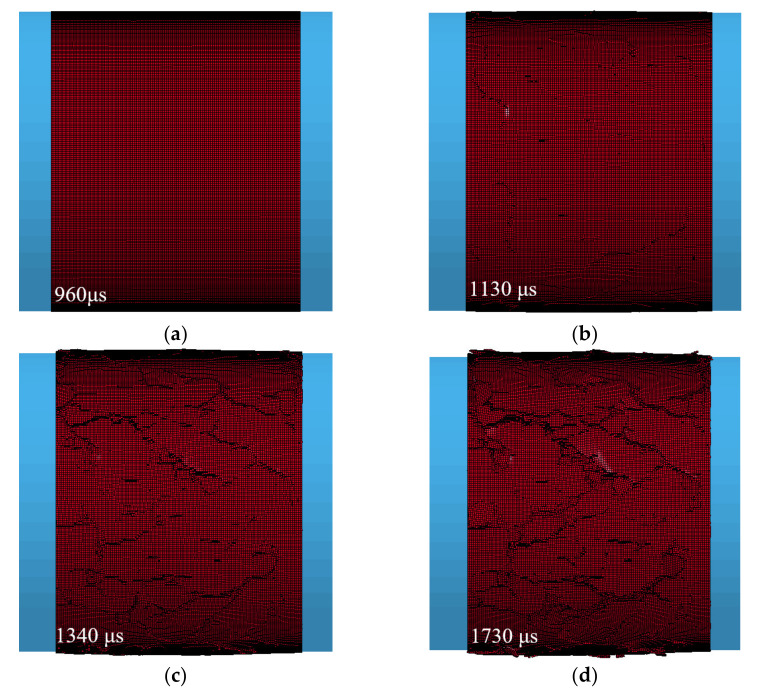
Deformation and failure process of concrete specimen at an impact velocity 15.33 m/s, (**a**) 960 μs; (**b**) 1130 μs; (**c**) 13400 μs; (**d**) 1730 μs.

Generally, numerical predictions require validation of experimental results. According to the simulation results of SHPB, it is a common way to compare the reflection wave, transmission wave and the deformation and failure process of the specimen obtained by experiment and numerical calculation [41]. In order to verify the validity of the mesoscale model and its constitutive parameters, parameters in Table 1 and Table 2 are used in this paper to simulate the concrete SHPB test conducted by Lv et al. [30]. As shown in Figure 6, the numerical simulation waveform curves with impact velocities of 15.33 m/s and 18.58 m/s are obtained and compared with the test results. The deformation and failure process of concrete at impact velocity of 15.33 m/s is shown in Figure 7.

In Figure 6, the simulation results at the two impact velocities both reproduce the phenomenon of the “double peak” of reflected wave and wave tail oscillation in the experiment results [4]. Lv [4] believed that the “double peak” phenomenon of reflected wave is caused by the three-phase mesoscopic structure of concrete material. After the incident wave enters the concrete specimen through transmission, the ITZ layer with the lowest strength is destroyed at first, and the interface between aggregate and mortar appears to fracture, then the first reflected wave peak appears. As the specimen is compacted, the mortar and aggregate contact directly and the stress wave continues to pass through. With the development of dynamic fracture in the specimen, the second reflection wave peak is formed. The characteristics of dynamic response behaviors of concrete material are well described by the establishment of a three-dimensional mesoscopic structure model. Figure 7 shows the deformation and failure process of concrete specimen. Under the impact loading, the specimen first produces an annular compression and expansion. And the surface elements reach the failure threshold and are deleted gradually, which finally causes the collapse of the specimen. The numerical results agree well with the experimental results in terms of the reflected wave, transmitted wave and the deformation and failure process of specimen. The specific difference between the reflected wave peak values and the crack distribution on the specimen surface are caused by the inevitable randomness, such as the aggregate shape and random distribution. In conclusion, the selected parameters of constitutive model and the three-dimensional mesoscopic structure model established in this paper is reliable and effective.

## 4. Simulation of Dynamic Splitting

Due to the low tensile property of concrete materials, the main failure form of concrete structures is tensile failure, and the tensile strength is an important material parameter of brittle materials. In this section, material parameters verified by rationality are mainly used to conduct numerical simulation of dynamic splitting on the mesoscopic finite element model of real aggregate established in Section 3.1. Stress response characteristics and fracture process of concrete are analyzed, and the damage mechanism of concrete is deeply understood. The impact of the tensile strength of three-phase mesoscopic structure on the tensile strength and energy of concrete is discussed.

### 4.1. Stress Variation Characteristic

**Figure 8:**
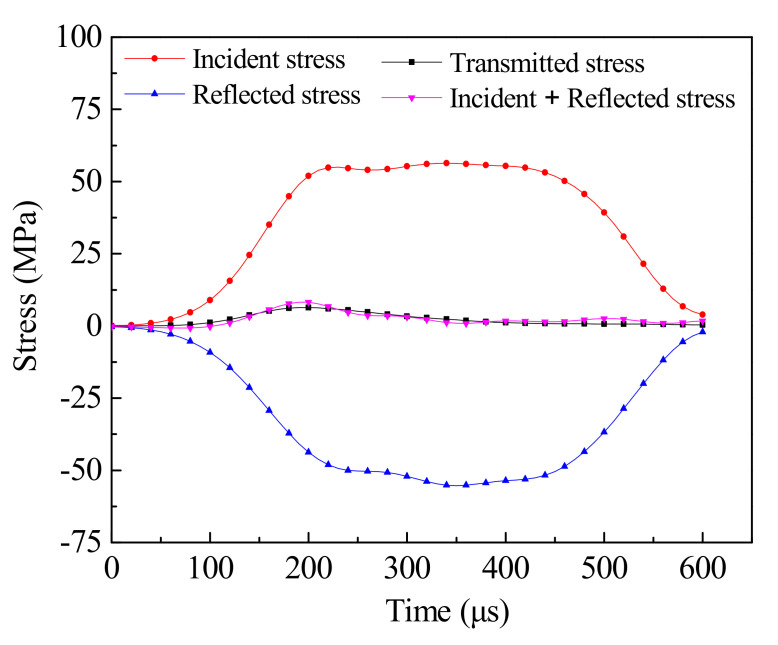
Verification of stress equilibrium.

According to the literature [30], the measured incident waves with the peak value of 33.15 MPa, 56.37 MPa, 80.54 MPa and 112.74 MPa were applied on the end face of the incident bar, and the rising stage was 170 µs, the peak duration was 198 µs, and the decrease time was 208 µs. The axial strain history curves of the elements at the center of the SHPB incident and transmission bars are extracted respectively, and the compressive strain rates and strains of the specimens under different incident waves can be obtained by using Equations (1) and (2) [12]. From the results of photo-elastic experiments, Gomez et al. [42] concluded that when a specimen experienced stress equilibrium, its dynamic stress distribution is similar to that of splitting stress under static load. However, both elastic analysis and photo-elastic experiments are based on homogeneous materials, while concrete is not uniform at the mesoscale. Guo [10] established, through elastoplastic analysis and experimental measurement, that once the stress equilibrium is reached, the dynamic tensile stress distribution at the center of the heterogeneous concrete specimen is similar to that of the homogeneous material, which can be estimated by static-elastic analysis. Figure 8 shows the stress balance verification of the wave in the propagation process. According to the one-dimensional stress wave principle and the assumption of stress uniformity, the stress at the incident end should be the same as that at the transmission end. If the superposition result of incident wave and reflected wave is equal to that of transmitted wave, it indicates that the specimen is in stress equilibrium state. It can be seen from Figure 8 that the transmitted stress is very close to the sum of the incident stress and the reflected stress, which can prove that the specimen is in a state of dynamic equilibrium during the whole process of stress wave propagation. Therefore, the dynamic tensile strength can be obtained from Equation (3).

(1) $$\dot{\varepsilon }(t)=\frac{C_{0}}{D}[\varepsilon_{i}(t)-\varepsilon_{r}(t)-\varepsilon_{t}(t)]$$

(2) $$\varepsilon (t)=\frac{C_{0}}{D}\int_{0}^{t}\left[\varepsilon_{i}(t)-\varepsilon_{r}(t)-\varepsilon_{t}(t)\right]\mathrm{dt}$$

(3) $$\sigma_{t}(t)=\frac{2AE}{\pi DL}\varepsilon_{t}(t)$$

where *έ*(*t*)*, ε*(*t*) and *σ_t_*(*t*) are the are the strain rate, strain, and stress of the specimen; *A* is the cross section area of the compression bar; *E* and *C*_0_ are the compression bar material elastic modulus and wave velocity, respectively; *ε_i_*(*t*), *ε_r_*(*t*), and *ε_t_*(*t*) are the incident strain, reflected strain, and transmitted strain of a certain time *t*, respectively; D and L are the diameter and thickness of the specimen, respectively; and *t* is the duration of the stress wave.

**Figure 9:**
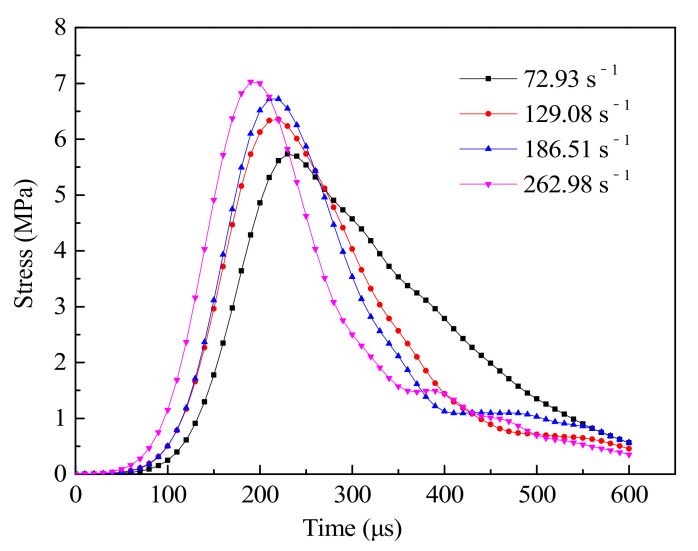
Tensile stress history curves.

Figure 9 shows the tensile stress history curves under different strain rate. The figure shows that the dynamic tensile strengths of concrete under different strain rate, changing over time, are similar, but with the increase of strain rate, the dynamic tensile stress of concrete also increasing. This shows that concrete is a typical strain rate related materials, the dynamic tensile strength will improve with the increase of strain rate. The higher tensile properties of specimens under high strain rates are generally considered to be related to the process of crack propagation and energy change under dynamic load. In general, the energy required for crack formation is greater than that needed for crack development, and the failure of concrete specimens tends to propagate along the ITZ interface (the lowest strength interface) [43]. However, at high strain rates, internal cracks do not have enough time to develop. Before the final failure formed, only a few microcracks attempt to expand into macro cracks, but more microcracks are formed and developed. Moreover, the energy input rate increases with the increase of strain rate. Concrete specimens absorb a large amount of energy at high strain rate and fail to release it in short time, which ultimately leads to the increase of tensile strength of concrete with the increase of stress rate. At the same time, it can be seen that with the increase of strain rate, the stress of concrete starts to rise in advance and reaches the peak strength in advance, and the time required for concrete to bear its load to the point of failure is reduced. The main reason for this phenomenon is that with the higher amplitudes of the loading incident wave, the strain rate is higher, and the more energy is input to the specimen per unit of time. The increase of the conversion rate of input energy increases the rate of change in the stress and deformation of concrete with time, that is, the time required for the specimen from loading to failure is shortened [44]. According to the stress descending section in the figure, it can be found that with the increase of strain rate, the steeper the stress descending section of the specimen becomes, and the decreasing speed increases. The main reason is that with the increase of strain rate, the energy stored in the sample increases when the peak stress is reached, and the energy release rate also increases during unloading, thus increasing the rate of stress decline.

### 4.2. Mesoscopic Failure Process of Concrete

**Figure 10:**
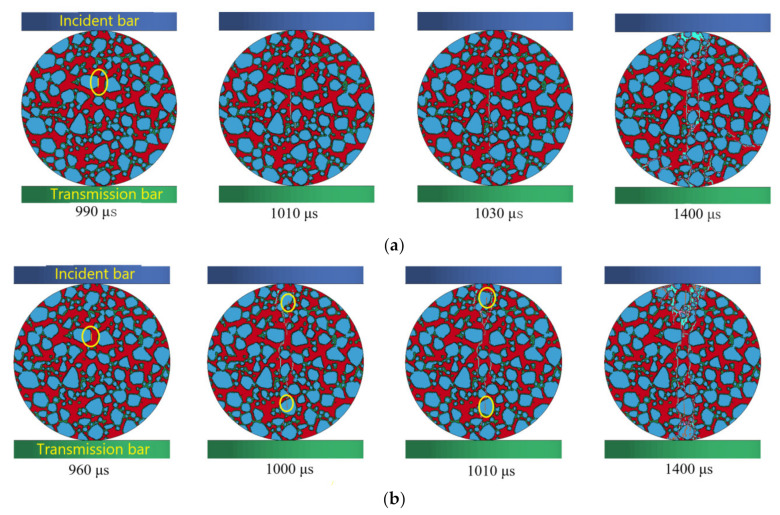
Failure process of concrete under different strain rates (**a**) 72.93 s^−1^; (**b**) 186.51 s^−1^.

For an effective dynamic splitting test, the specimen should first fracture along the loading direction near the center of the disc. When the unshaped pulse wave is loaded, the first failure of the specimen usually occurs at the incident end after the incident wave reaches the bar/specimen interface. Soon after, the transmission end of the specimen will also be damaged. In the dynamic splitting test, the stress pulse propagates inside the disc specimen, causing tensile stress to be generated in the center of the specimen perpendicular to the direction of the loading surface, which leads to the crack initiation near the center of the concrete specimen, and the crack propagation along the loading direction. Finally, the crack coalesces and destroys the specimen [45]. Figure 10 shows the dynamic fracture failure process of concrete at different strain rates, in which there are four graphs at each strain rate, representing the crack initiation, crack development, peak stress time and the sample section after the final failure respectively. It can be found that, because the incident waves are measured after pulse molding, the phenomenon of crack initiation at the end is avoided, and because the strength of ITZ is low, the crack initiation of the concrete mesoscopic model all occurs entirely on the ITZ near the center of the specimen, and the crack propagates along the loading ends on both sides. This phenomenon is consistent with the dynamic splitting test process reported by Xia [46], with a high-speed camera, which satisfies the basic hypothesis of the dynamic splitting test, indicating that it is feasible to calculate and analyze the numerical results through Equation (3). Combined with Figure 10a,b, it can be found that when the strain rate is 72.93 s^−1^, the crack initiation time and peak stress time of the sample are 990 µs and 1030 µs, respectively. When the strain rate is 186.51 s^−1^, the crack initiation time and peak stress time are 960 µs and 1010 µs, respectively. This shows that if the strain rate, crack time and peak stress reached moments earlier is higher, the stress of the specimen conversion rate is higher, mainly because of the higher strain rate, and the stress increases more quickly over time, which causes the crack moment to occur earlier and crack propagation to also take place ahead of time, and energy released by the propagation of the crack causes the specimen to reach peak stress at an earlier point in time. When the concrete is under the peak stress, the cracks in the concrete have not been connected, and before the cracks are connected, there are few cracks in the concrete, and there are lots of main cracks. It can be seen that the dynamic tensile strength of concrete is less affected by the number of cracks, and the number of cracks is determined by the mechanical properties after the peak stress. The crack penetration moment is greatly affected by the impact velocity, and the force of concrete at this time increases with the increase of the impact velocity. Thereafter, the excess force will lead to the development of secondary cracks of concrete and the rapid splashing of fragments.

It can be found that at the same time, as a result of the aggregate strength and elastic modulus are higher than mortar and ITZ, when strain rate is 72.93 s^−1^, in the process of failure, most of the cracks in the sample bypass the aggregate and propagate through the mortar and ITZ, few cracks can propagate through the aggregate directly. Crack arrest occurs when the crack expands to the aggregate, and sufficient energy continues to be stored at the crack tip before further propagation failure, which indicates that the aggregate hinders the propagation of concrete cracks under dynamic load. However, when the strain rate increases (186.51 s^−1^), the energy release capacity of concrete increases, leading to the increase of energy stored at the crack tip and the increase of crack propagation directly through the aggregate. It can be seen that the propagation mode of crack is related to the energy stored at the crack tip [44]. When the strain rate is low, the energy input to the crack tip per unit of time is low, and the energy stored at the crack tip is less, so the crack propagation direction is to bypass the aggregate and propagate along the weak interface element. When the strain rate is high, the energy input to the crack tip per unit of time is high. As the energy cannot be fully released at one time, the crack does not crack in time, and the energy stored at the crack tip increases, which is enough to make the aggregate crack directly. Therefore, the crack will directly pass through the aggregate and expand in a direction parallel to the loading direction.

### 4.3. Effect of Material Strength of Each Phase on Mechanical Properties of Concrete

As a three-phase composite material composed of mortar, aggregate and ITZ, the material strength of each component is different, so the dynamic failure process of concrete is greatly influenced by each component. The three-dimensional mesoscale model can adjust the material strength of three-phase components, analyze the sensitivity of material strength of each component, study the role of each component in concrete, and further explore the methods to improve the dynamic tensile strength of concrete. The tensile strength of mortar, aggregate, and ITZ was multiplied by 2 and 0.5, respectively, that is, the tensile strength of each component increased by 100% and decreased by 50%. Under the loading condition of strain rate of a 72.93 s^−1^ strain rate, the influence of tensile strength of each composition in concrete on macroscopic mechanical properties was studied. 

**Figure 11:**
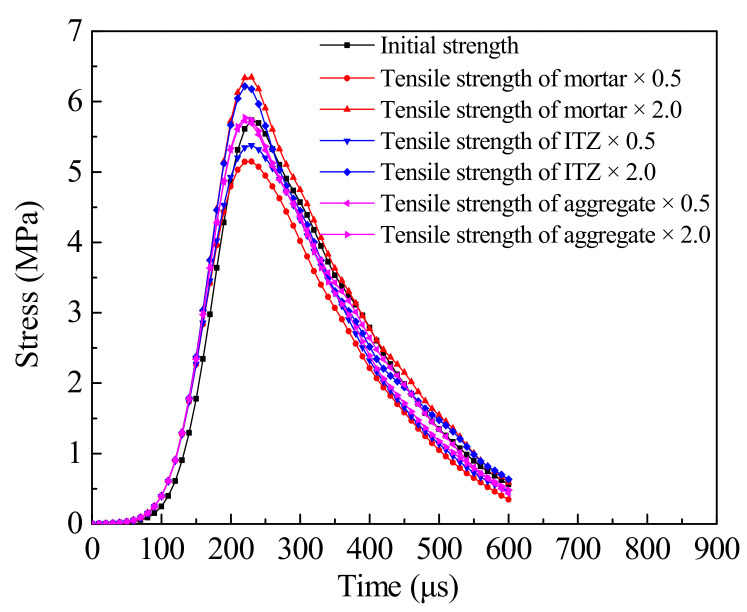
Tensile stress history curves under different parameters.

The tensile stress history curves with different tensile strength of three-phase composition are plotted in Figure 11. It can be seen that the peak stress of concrete is 5.73 MPa when the initial material parameters are used. The tensile strength of aggregate increases by 100% and decreases by 50%, the peak stress of concrete is the same as when the initial parameters are adopted, indicating that the change of tensile strength of aggregate has little effect on the dynamic tensile strength of concrete. When the tensile strength of mortar and ITZ increased by 100%, the dynamic tensile strength of concrete was 6.34 MPa and 6.21 MPa, respectively, representing increased by 10.6% and 8.4%. When the tensile strength of mortar and ITZ is reduced by 50%, the dynamic tensile strength of concrete is 5.15 MPa and 5.38 MPa, respectively, which represents reductions of 10.12% and 6.11%. It can be seen that the dynamic tensile strength of concrete is the most affected by the tensile strength of mortar, followed by ITZ, and is almost unaffected by aggregate. The main reason is that mortar, as the main component material of concrete, accounts for the largest proportion and connects the whole concrete specimen, so the material strength of mortar has the greatest influence on the peak stress of concrete. The lower the tensile strength of mortar, the lower the overall strength of concrete, and the easier it is for materials to be damaged. Aggregate particles are dispersed and isolated, and the tensile strength of aggregate is far higher than that of ITZ and mortar. Even if it is reduced by 50%, it is still higher than that of mortar and ITZ. Cracks mainly spread along ITZ and mortar, which makes aggregate play more role in storing energy, so aggregate has less influence on concrete strength. ITZ is the weak part of concrete, and cracks often start from this area. When the strength of ITZ decreases, cracks are more likely to occur and propagate along ITZ, and concrete is more likely to be destroyed. Its influence on the tensile strength of concrete is between that of mortar and aggregate.

**Figure 12:**
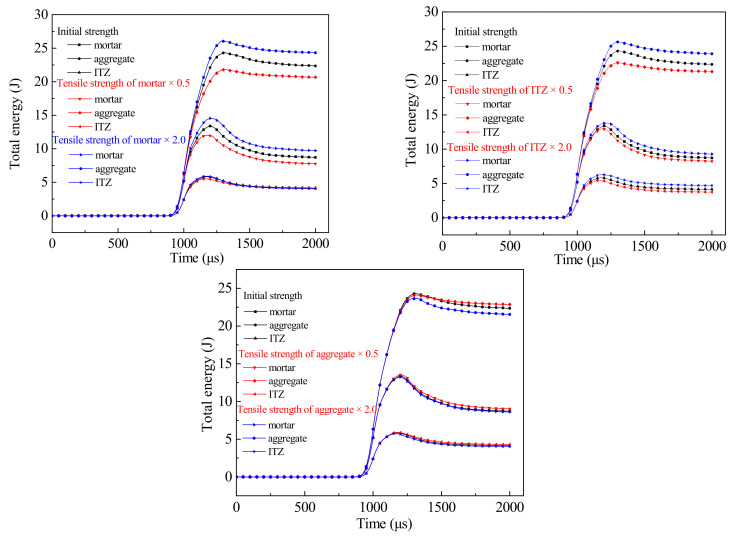
Energy history curves of different parameters.

The dynamic failure process of concrete is always accompanied by the variation of energy. The energy of the concrete specimen at each moment can be drawn from the numerical results. The effect of tensile strength on the energy of each component is shown in Figure 12. It can be seen from the figure that no matter whether the tensile strength of mortar, ITZ and aggregate increases or decreases, the energy of the aggregate is always the highest among all components, followed by mortar, and the energy of ITZ is the smallest. The main reason that the failures aggregate elements are much smaller than those of mortar and ITZ. Most aggregate particles remain intact and have high energy storage capacity. In the process of stress wave propagation, the input energy is stored in the aggregate. Although mortar is the largest part of the concrete specimen, there are more elements in the energy dissipation failure, which cannot continue to transfer and store energy, so the energy is less than that of the aggregate. The volume of ITZ itself in concrete specimens is relatively low, and it is easy to crack as a weak interface, and the failure volume is the highest, so the energy in ITZ is the smallest. As the tensile strength of mortar increases by 100%, the energy of ITZ in three-phase components changes little, and the energy of aggregate and mortar increases by 7.4% and 8.7% respectively. When the tensile strength of mortar decreases by 50%, the energy of the aggregate and mortar reduces by 10.3% and 10.5% respectively. When the tensile strength of ITZ increases by 100%, the energy of aggregate and mortar increases by 5.51% and 3.29% respectively. When the tensile strength of ITZ decreases by 50%, the energy of the aggregate and mortar decreases by 7.04% and 2.99% respectively. When the tensile strength of aggregate increases by 100% and decreases by 50%, the energy of three-phase components is basically equal to that of the initial parameters. It can be seen that the mortar material has the greatest influence on the energy, followed by ITZ, which is almost not affected by the aggregate strength, and this law is consistent with the influence of each component on the tensile strength of concrete. The reason is that the tensile strength of aggregate decreases by 50%, and it still has high strength. Failure volume of aggregate is small, and it can continue to store energy. While the input energy constant, and it cannot reach the limit of aggregate storage energy. Therefore, changing the aggregate strength has little effect on the energy change of concrete. The mortar matrix has the largest volume, and the higher the strength is, the more difficult the crack is to expand in the mortar matrix, and the specimen is more difficult to damage. More energy will continue to be stored in the concrete. On the contrary, the energy consumption per unit of volume of crack initiation and propagation decreases, and it is easy to expand in the mortar matrix. Therefore, changing the strength of the mortar matrix has a great impact on the change of concrete energy.

Generally speaking, the strength of the concrete structure is limited by the weakest part within it, and the weakest part should also be considered when improving the strength of a structure. Through sensitivity analysis of the tensile strength of each component, it can be seen that the ITZ has the lowest strength and is most likely to fail, which affects the energy release process of concrete and further affects its tensile strength. Mortar bypass through the entire specimen and has the largest volume ratio, which can directly affect the energy absorption capacity of concrete through its own strength. Therefore, in the dynamic test, the tensile strength of the concrete structure is primarily determined by the mortar and ITZ. In practical engineering construction, the most direct way to improve the dynamic tensile strength of concrete is to use some methods to improve the tensile strength of mortar matrix, and the high bond strength of ITZ is also an important guarantee for high tensile strength of concrete. In order to improve the dynamic tensile strength of concrete, the strength of mortar should be first considered in the preparation of concrete, and then the ITZ strength should be ensured in the preparation process. The tensile strength of aggregate has little effect on the dynamic strength of concrete, only slightly more than that of mortar.

## 5. Conclusions

Concrete was regarded as a three-phase material composed of aggregate, mortar and the interfacial transition zone between them. Based on the fractal characteristics of concrete aggregate, a three-dimensional mesoscopic structure model was established. The mesh mapping method was used to establish a numerical model, and the effect of strain rate on the dynamic indirect tensile strength and the progressive failure process, at the mesoscopic scale, was studied by simulating the dynamic splitting under SHPB loading. By changing the material parameters, the influence of the tensile strength of three-phase components on its dynamic indirect tensile strength is analyzed. And the main conclusions are as follows:

1. Compared with spherical and convex polygon aggregates, we have obtained the fractal characteristics of realistic aggregates by the image processing algorithm, and based on this approach, generated a 3D mesoscopic geometric model that is more consistent with the realistic aggregate. Furthermore, the grid mapping method is used to generate the numerical model. By comparing the reflection and transmission waveform and failure process of concrete dynamic compression experiment, the validity of the mesoscopic structure model of concrete is verified.
2. Based on the 3D mesoscopic model, we simulate the dynamic splitting of concrete and reproduce the rate correlation of its tensile strength, i.e., the larger the amplitude of incident wave is, the larger the amplitude of transmitted wave is. As can be seen from the dynamic failure process obtained by simulation, the failure occurs at the interfacial transition zone, then extends to the mortar, and the aggregates rarely fail. However, the increase of strain rate intensifies this process. When the strain rate increases from 72.93 s^−1^ to 186.51 s^−1^, a large number of aggregate elements are deleted due to reaching the failure threshold.
3. The variation of tensile strengths of each phase component have the same effect on the dynamic tensile strength and energy of concrete. The dynamic tensile strength and energy of concrete are most affected by the tensile strength of mortar, followed by the ITZ, but the tensile strength of aggregate has almost no effect.

## Figures and Tables

**Table 1:** Parameters of mortar.

Parameter	Value	Parameter	Value
Density ρ (kg/m^3^)	2200	Compressive damage scaling parameter	1.6
Poisson ratio	0.19	Unit conversion factor for stress	145
Compressive strength (MPa)	50	Tensile damage scaling exponent	1.35
Uniaxial tensile strength (MPa)	4.0	Residual failure surface coefficient	236.6
Maximum shear failure surface parameter *A*_0_	1.47 × 10^−4^	Initial yield surface coefficient	515.0
Maximum shear failure surface parameter *A*_1_	0.446	Maximum shear failure surface parameter *A*_2_	161.6

**Table 2:** Parameters of interfacial transition zone (ITZ).

Parameter	Value	Parameter	Value
Density ρ (kg/m^3^)	1800	Compressive damage scaling parameter	1.6
Poisson ratio	0.19	Unit conversion factor for stress	145
Compressive strength (MPa)	35	Tensile damage scaling exponent	1.35
Uniaxial tensile strength (MPa)	3.2	Residual failure surface coefficient	338.0
Maximum shear failure surface parameter *A*_0_	1.03 × 10^−4^	Initial yield surface coefficient	735.7
Maximum shear failure surface parameter *A*_1_	0.446	Maximum shear failure surface parameter *A*_2_	230.9

**Table 3:** Holmquist-Johnson-Cook (HJC) model parameters of aggregate.

Parameter	Value	Parameter	Value
Density ρ (kg/m^3^)	2660	Normalized maximum strength SFMAX	4
Shear modulus G (GPa)	21.5	Crushing pressure *P*_crush_ (MPa)	53
Normalized cohesive strength *A*	0.9	Crushing volumetric strain *μ*_crush_	0.0012
Normalize pressure hardening *B*	2	Locking pressure *P*_lock_ (MPa)	800
Pressure hardening exponent *N*	0.65	Locking volumetric strain *μ*_lock_	0.01
Strain rate coefficient *C*	0.1	Damage constant *D*_1_	0.08
uniaxial compressive strength *f*_c_ (MPa)	160	Damage constant *D*_2_	1.0
Maximum tensile pressure *f*_t_ (MPa)	10	Pressure contant *K*_1_ (MPa)	1.4 × 10^4^
Amount of plastic strain before fracture EFMIN	0.01	Pressure contant *K*_2_ (MPa)	−20 × 10^5^
Maximum principal strain at failure MXEPS	0.1	Pressure contant *K*_3_ (MPa)	25 × 10^5^

## Data Availability

The data presented in this study are openly available in [Figures 17 and 19] at [10.1016/j.conbuildmat.2017.11.094], reference number [30].
